# Triage under pressure: Nursing students’ learning and preparedness in a code orange simulation using CTAS

**DOI:** 10.1371/journal.pone.0360652

**Published:** 2026-10-30

**Authors:** Vanessa Silva e Silva, Magdalena Dobosiewicz, Karina Tamy Kasawara, Emily Bot, Sylas Coletto, Rebecca Larocca, Shelley Oakes, Tori McDowall, Dawn Prentice, Amina Silva

**Affiliations:** Nursing, Brock University, St. Catharines, Ontario, Canada; University of Zagreb School of Medicine: Sveuciliste u Zagrebu Medicinski fakultet, CROATIA

## Abstract

**Background:**

Preparing nursing students for mass casualty incidents requires more than foundational knowledge; it demands the ability to apply triage principles under pressure. While simulation-based education is widely used, there is limited evidence examining how students integrate established triage frameworks, such as the Canadian Triage and Acuity Scale (CTAS) and Glasgow Coma Scale (GCS), within high-acuity disaster contexts. This study evaluated nursing students’ knowledge application, clinical prioritization, and perceived preparedness following a Code Orange simulation.

**Methods:**

cross-sectional survey was conducted with undergraduate nursing students (n = 26) following participation in a high-fidelity Code Orange simulation. The survey included multiple-choice knowledge items assessing CTAS and GCS application, Likert-scale measures of perceived knowledge, confidence, and preparedness, and open-ended questions exploring student experiences. Quantitative data were analyzed using descriptive statistics and Chi-square tests, while qualitative responses underwent content analysis.

**Results:**

Participants demonstrated strong knowledge of CTAS and GCS principles, with most correctly identifying triage priorities and neurological assessment parameters. Variability was observed in students’ ability to distinguish between CTAS Levels 2 and 3, with recognition of vital signs as a key differentiator (χ² = 57.18, p < .001). Likert-scale responses indicated high levels of perceived preparedness, confidence, and knowledge following the simulation. Qualitative findings highlighted the importance of realism, cognitive demand, and teamwork in shaping learning, with students emphasizing the value of applying theoretical knowledge in a dynamic, high-pressure environment.

**Conclusion:**

Findings suggest that high-fidelity disaster simulations extend beyond reinforcing baseline knowledge by promoting the application of triage decision-making in complex clinical contexts. Integrating structured triage frameworks within immersive simulation may enhance preparedness for mass casualty response, particularly in areas requiring nuanced clinical prioritization. These results underscore the role of simulation in bridging the gap between theoretical knowledge and real-world emergency decision-making.

## Introduction

Disaster preparedness is an essential component of nursing education, as nurses play a critical role in mass casualty incidents (MCIs) and must be able to rapidly assess, prioritize, and manage patient needs in high-acuity environments [1,2]. The Canadian Triage and Acuity Scale (CTAS) provides a standardized framework for categorizing patient acuity and remains a key competency in emergency and disaster nursing across Canada [3]. Despite its importance, the integration of disaster nursing content within undergraduate curricula remains inconsistent, resulting in variable exposure to disaster-preparedness training and potential gaps in readiness for clinical practice [4,5].

International literature further highlights a limited body of work describing the design and evaluation of mass casualty simulations in nursing education, particularly within Canadian contexts [2,5]. Emerging evidence underscores the ongoing need for accessible, practice-oriented educational strategies that not only build knowledge but also support the application of clinical judgment under pressure [6,7]. Simulation-based disaster education, particularly using high-fidelity scenarios and standardized patients, has been shown to enhance disaster nursing competencies, critical thinking, and triage performance among undergraduate students [6,7]. As such, simulation-based learning is increasingly recognized as an evidence-informed approach for developing clinical decision-making, communication, and teamwork skills in complex and rapidly evolving care environments.

High-fidelity simulation offers a psychologically safe yet immersive environment in which students can practice rapid decision-making without risk to patient safety. he National League for Nursing (NLN) Jeffries Simulation Theory emphasizes the importance of structured pre-briefing, realistic scenario design, and facilitated debriefing in promoting deep learning and clinical reasoning in complex situations such as MCIs [8]. In the context of disaster education, simulation uniquely enables learners to experience the cognitive load, uncertainty, and time pressure inherent to triage, factors that are difficult to replicate in traditional classroom settings [7]. These experiential elements have been associated with improved knowledge retention, enhanced teamwork behaviours, and greater transferability of skills to real-world practice [9,10].

To address these gaps, a high-fidelity Code Orange disaster simulation was developed incorporating professional and volunteer actors, environmental cues, and realistic scenario progression to replicate the complexity of an MCI. This study aimed to evaluate nursing students’ application of CTAS and GCS principles and their perceived preparedness following participation in the simulation using a cross-sectional survey design.

## Materials and methods

### Study design and setting

A cross-sectional survey design incorporating a convergent (concurrent) mixed-methods component [11], which quantitative (multiple-choice and Likert-scale) and qualitative (open-ended) data were collected at the same pre- and post-simulation time points and analyzed in parallel, to evaluate nursing students’ perceptions, self-reported confidence, and perceived learning outcomes following participation in a Code Orange (mass casualty incident) simulation. This design involves the collection of data at a single point in time and is appropriate for capturing participants’ immediate reactions and reflections after an intervention such as simulation-based training [11].

The cross-sectional approach was selected to provide a snapshot of students’ experiences and to assess the perceived effectiveness of the simulation in enhancing knowledge, clinical decision-making, and preparedness for disaster response. Data were collected immediately before and following the simulation and structured debriefing to minimize recall bias and ensure that responses reflected participants’ most recent experiences.

This design is commonly used in educational and simulation research, as it allows for efficient data collection from a defined cohort without the need for longitudinal follow-up. While causal relationships cannot be established, the design is well suited for exploratory evaluation and for identifying trends in student perceptions that may inform future curriculum development and simulation design.

### Participants

All undergraduate students enrolled in a concurrent bachelor’s and master’s program in nursing (n = 34) were invited to participate by the a faculty member who was not involved in this study (DAB) on May 15, 2025 and the participation in the activity and data collection was done on May 16, 2025. Eligibility criteria included active course enrollment, participation in the simulation activity, and provision of written informed consent. The study was voluntary and ungraded. In total, seven faculty and simulation laboratory coordinators facilitated the simulation, and eight undergraduate nursing student volunteers served as patient actors. Student volunteers were provided with standardized scripts and received training on role expectations, appropriate responses for each clinical context, and the use of moulage to ensure consistency and realism across scenarios. Of the 34 eligible students, 34 completed the pre-test survey, 26 completed the post-test, and 23 provided fully matched pre- and post-test responses (a matched participation rate of approximately 74% of eligible students). Because participation in the research component was voluntary, the possibility of self-selection bias cannot be excluded; demographic or academic characteristics of non-participating or non-matched students were not collected and could not be compared to participants.

### Simulation procedure

The Code Orange (MCI) simulation took place on campus at Brock University between May and August 2025. The intervention simulated an MCI (Code Orange) involving patients with varying levels of acuity. Using mannequins and trained actors portraying injured victims, the exercise included visual and auditory cues (screaming, sirens, simulated wounds, and smoke) to replicate realistic environmental stressors. Students performed key clinical assessments, including level of consciousness, however, vital signs were provided rather than simulated to ensure alignment with assigned CTAS levels. The scenario lasted approximately 30 minutes, preceded by a structured pre-briefing involving orientation of hospital codes, emergency preparedness, triage system and personal safety, and followed by 60 minutes debriefing session to support reflection and emotional processing.

The scenario was introduced to students during the pre-brief stage for the Code Orange simulation. Students were asked to close their eyes if comfortable and imagine the following scenario: *“A sound of an explosion went off on the computer speakers in the lab and the following excerpt was read: “During your shift, you feel the hospital walls, and floor rumble. You run to the closest window and look outside and see paramedics, firefighters, police and others rushing to the scene in the hospital parking lot. An announcement is made over the hospital speakers “CODE ORANGE stage 1 parking lot C, CODE ORANGE stage 1 parking lot C, CODE ORANGE stage 1 parking lot C” and you hear from staff that there was an explosion. You report to your designated area and are informed the police, Chemical, Biological, Radiological, Nuclear, Explosive team (CBRNE), Hazardous Materials team (HAZMAT) and fire have secured the scene. You are assigned to be the external triage nurse and will be accompanied by the firefighters to the parking lot due to the need for further resources. There you will find six patients who have been injured by the explosion.”*

Students worked in interprofessional teams to assess patients and assign appropriate CTAS levels. Each group rotated through multiple victim scenarios, communicating with victims and each other, calculating GCS scores and discussing and documenting triage priorities collaboratively. Each group had three minutes, the Code Orange facilitator (MD) cued for all groups to rotate to the next victim scenario. The debriefing phase was completed in simulation lab setting and was facilitated by an experienced ER nurse using the PEARLS framework [12] and focused on reflective discussion of GCS/CTAS scoring, patient prioritization, and resource allocation. Students were informed of Code Orange response roles, communication procedures with media, and operational considerations such as bed availability, staffing, and hospital entry security. Then, the debriefing was followed by a structured reflective framework to consolidate learning outcomes and emotional responses (Appendix A).

The intervention consisted of a full-scale simulated Code Orange scenario adapted from a 2023 tabletop exercise [13]. Six standardized patient scripts were created based on MCI injury patterns and reviewed by clinical experts for accuracy in GCS and CTAS scoring (Table 1 and and Appendix B). The scenario incorporated moulage, ambient sound, fog effects, and safety protocols to enhance fidelity. Students assessed each “patient,” assigned CTAS categories, and documented rapid clinical findings. A structured debrief was facilitated by an emergency nursing expert following best practices in simulation pedagogy. Details on the actual development of the activity and resources will be shared in a separate publication.

**Table 1:** Mass Causality Incidents (Code Orange) simulation scenarios.

Patient 1 – Debris in Eyes	Young adult with severe eye pain and vision difficulty but stable overall (CTAS 3).
Patient 2 – Semiconscious Burn Victim	Older teen with major burns, unstable vitals, and decreased consciousness requiring immediate intervention (CTAS 1).
Patient 3 – Hearing Loss and Bloody Nose	Adult with sudden hearing loss and mild facial injuries but stable vitals (CTAS 4).
Patient 4 – Leg Impalement	Older adult with a severe leg impalement and signs of shock needing emergent care (CTAS 1).
Patient 5 – Small Abrasions to Hands	Adult with minor abrasions who is emotionally distressed but medically stable (CTAS 5).
Patient 6 – Deceased Patient	Adult found unresponsive with no vital signs requiring resuscitation if resources permit (CTAS 1).

The event took place in a secured alleyway between campus buildings, selected and approved by university emergency management and facilities personnel. The simulation lasted approximately two hours, including a 30-minute pre-brief, 30-minute scenario, and 60-minute debrief.

### Instruments and data collection

Data were collected using Qualtrics surveys hosted on secure University servers. Participants generated a unique, anonymous identifier to link their pre- and post-simulation responses while maintaining confidentiality. The pre-test survey was administered 24–48 hours prior to the simulation, and the post-test survey was completed immediately following the debriefing session. Each survey required approximately 15 minutes to complete.

The questionnaire consisted of three components. The first section captured demographic information, including age range and gender. The second section assessed knowledge through 10–15 multiple-choice and case-based items evaluating CTAS and GCS criteria, disaster triage principles, and clinical prioritization. The third section measured self-confidence and perceived preparedness using 8–10 Likert-scale items focused on triage decision-making, communication, and performance under pressure. We also included open-ended questions inviting participants to reflect on their learning, teamwork experiences, and suggestions for improving the simulation. The knowledge, confidence, and preparedness items were developed by the research team specifically for this study, aligned with course learning objectives and existing CTAS/GCS teaching content; the instrument had not undergone prior formal psychometric validation (e.g., a priori content validity indexing or internal consistency testing such as Cronbach’s alpha) at the time of this evaluation.

### Risk management and participant safety

The simulation involved exposure to realistic but potentially distressing stimuli, including loud noises, simulated injuries, and mock fatalities. To minimize risk, students participated in a structured pre-brief to prepare them for the intensity and emotional demands of the scenario. In advance of the exercise, students had participated in prior laboratory sessions that allowed them to practice relevant assessment tools, including CTAS and GCS, in lower-acuity patients scenarios. On-site safety measures were implemented, including the presence of trained personnel, access to shaded areas, and availability of hydration. The broader university community and nearby businesses were notified in advance of the simulation, and the area was clearly delineated and temporarily restricted to prevent interference by bystanders and to ensure public safety. Participation was voluntary, and students were reminded that they could withdraw from the research component or the simulation activity at any point without penalty. A facilitated debriefing followed the scenario to support emotional processing, address any distress, and reinforce key learning outcomes. These safeguards ensured that educational fidelity did not compromise participants’ psychological or physical safety.

### Data analysis

Survey data were analyzed using a combination of descriptive, inferential, and qualitative analytic approaches. Quantitative data were imported into SPSS (Statistical Package for the Social Sciences) for analysis. Descriptive statistics, including frequencies and percentages, were used to summarize demographic characteristics, CTAS and GCS knowledge items, and Likert-scale measures of perceived knowledge, confidence, and preparedness. Associations among categorical variables were examined using Chi-square tests, with exact tests applied where cell counts were small. Likert-scale responses were analyzed descriptively to characterize overall response distributions. Statistical significance was set at p < 0.05. Because Likert-scale and knowledge-item responses were ordinal or categorical in nature, no assumption of normality was required; parametric tests such as paired t-tests were not used. Chi-square tests of independence (with Fisher’s exact test substituted where expected cell counts were below 5) were applied to compare the distribution of responses between pre- and post-test time points for each categorical/ordinal item.

Responses to open-ended questions were de-identified and analyzed using conventional content analysis. Data were managed in NVivo qualitative analysis software (QSR International, AU). An initial coder (VSS) conducted inductive coding to identify recurring concepts and patterns, followed by independent review of the coding framework by a second researcher (AS). Discrepancies were resolved through discussion and consensus to enhance analytic rigor. Analysis was iterative and concurrent with data familiarization to ensure depth and completeness of thematic representation. Two coders (KK and VSS) were involved: the initial coder developed the coding framework inductively, and the second coder independently reviewed all coded transcripts against the framework. Disagreements were resolved through discussion and consensus and consultation of a third member (AS) when needed, rather than through a quantitative intercoder reliability statistic (e.g., Cohen’s kappa); member checking with participants was not performed as part of this evaluation.

### Rigour and trustworthiness

In qualitative research, “trustworthiness” (credibility, dependability, confirmability, and transferability) is the established framework used to evaluate methodological rigour, analogous to reliability and validity in quantitative research [14]. The following trustworthiness strategies constituted the rigour measures implemented within the qualitative component of this cross-sectional study. Credibility was strengthened through analyst triangulation, with two reviewers independently coding the data and reaching consensus through discussion [14]. Dependability was enhanced by maintaining an audit trail of coding decisions, category revisions, and peer-review notes throughout the analytic process [15]. Confirmability was supported by using de-identified textual data and documenting reflexive notes during coding to minimize researcher bias [16]. Transferability was promoted by providing sufficient detail on data collection procedures, participant context, and analytic steps, enabling readers to determine applicability to other educational or simulation settings [17]. Together, these strategies ensured that the qualitative findings were systematically derived and methodologically robust.

### Ethical considerations

Ethical approval was obtained from the Brock University Health Science Research Ethics Board (#24-308). Participation in the research component was voluntary, and all data were de-identified. To prevent coercion, recruitment and consent were facilitated by a faculty member (DA) not involved in course teaching or grading who volunteered to introduce the study to the students. Students were informed of their right to withdraw at any time before data analysis without academic consequence. Students who chose not to participate still completed the simulation and evaluation as part of regular coursework but were excluded from the data analysis.

## Results

A total of 34 students completed the pre-test and 26 completed the post-test, with 23 participants providing matched pre- and post-test responses included in the quantitative analysis. Most participants were young adults between the ages of 18–24, and the majority identified as women (78%). A one-sample exact binomial test confirmed that the proportion of women was significantly higher distribution (p < .001).

Across the CTAS knowledge items (Table 2), participants demonstrated a strong baseline understanding of triage principles, with patterns of stability observed between pre- and post-test responses. Nearly all students correctly identified CTAS Level 1 as the highest priority level in both assessments. Similarly, when classifying a patient with chest pain, diaphoresis, and tachycardia, the majority of participants selected CTAS Level 2 at both time points, with no statistically significant difference detected (χ²(2, N = 23) = 0.33, p = .848).

**Table 2:** Perceived Knowledge of CTAS, GCS and Triage in Disaster Settings (n = 23; scale 1 = Strongly Disagree to 5 = Strongly Agree).

Item	Statement	Pre-test Median	Pre-test IQR	Post-test Median	Post-test IQR
Q1	Understands the purpose of CTAS	5	4–5	5	5–5
Q2	Can identify correct CTAS level from symptoms	4	3–4	4	4–5
Q3	Can differentiate CTAS Level 2 vs 3	3	3–4	4	4–4
Q4	Understands how CTAS is applied in MCIs	4	3–4	5	4.5–5
Q5	Confident assigning CTAS levels under pressure	4	3–4	4	4–5
Q6	Can prioritize patient care using CTAS in a disaster	4	3–4	4	4–5
Q7	Knows CTAS level for non-urgent conditions	5	4–5	5	5–5
Q8	Understands elements essential for effective triage	4	3–4	4	4–5
Q9	Understands components of the GCS	4	4–4	5	5–5
Q10	Confident assigning a GCS score	4	4–4	5	5–5
Q11	Can integrate GCS scoring into triage decisions	3	3–4	5	4.5–5

Students’ understanding of the difference between CTAS Levels 2 and 3 showed the only statistically significant shift. While “pain level” and “time to physician” remained the most commonly selected factors, more participants selected “vital signs” post-simulation, resulting in a significant change in response distribution (χ²(12, N = 23) = 57.18, p < .001). Classification of a stable patient requiring simple investigation or treatment remained consistent, with most participants selecting CTAS Level 5 in both tests and no significant change detected (p = 1.000). Similarly, participants consistently identified allocation of hospital funding as *not* a key element of effective triage with no meaningful difference between assessments (p = 1.000).

Knowledge of the GCS was uniformly strong, with all participants correctly identifying the three GCS components and the maximum score of 15 at both time points. Interpretation of a GCS score of 8 or below also remained stable, with most students selecting “severe brain injury” in both assessments and no statistically significant change detected (p = .500). Overall, these results indicate high baseline knowledge of CTAS and GCS principles, with the simulation influencing only one specific aspect—participants’ recognition of vital signs as a distinguishing factor between CTAS Levels 2 and 3. The high baseline level of knowledge observed may be attributable to prior simulation laboratory experiences focused on the GCS and the CTAS, as well as opportunities for students to practice these assessments with direct feedback from laboratory coordinators before participation in the Code Orange exercise. Providing structured education and practice before the Code Orange was intentionally aligned with current course objectives and was essential to supporting learner confidence and readiness, as well as promoting psychological safety, for students engaging in a high-intensity disaster simulation.

A comparison of pre- and post-test responses demonstrated a clear improvement in participants’ perceived knowledge of the Canadian Triage and Acuity Scale (CTAS), the Glasgow Coma Scale (GCS), and triage procedures in disaster settings (Table 3). As illustrated in the table above, post-test distributions shifted toward higher Likert ratings across all eleven items, indicating stronger agreement with statements reflecting understanding and confidence. Prior to the simulation, responses clustered primarily around “Agree” (4) with some variability; following the intervention, the majority of participants selected “Strongly Agree” (5) for nearly every item. The largest observed gains were seen in participants’ understanding of how CTAS is applied in mass casualty incidents, confidence in assigning CTAS levels under pressure, and ability to integrate GCS scoring into triage decisions. These findings suggest that the Code Orange simulation effectively enhanced both cognitive understanding and perceived self-efficacy in triage decision-making.

**Table 3:** Self-Confidence and Perceived Preparedness (n = 23; scale 1 = Strongly Disagree to 5 = Strongly Agree).

Item	Statement	Pre-test Median	Pre-test IQR	Post-test Median	Post-test IQR
Q1	Confident applying CTAS in a real-world scenario	4	3–4	4	4–4.5
Q2	Can accurately distinguish CTAS levels during triage	4	3–4	4	4–4
Q3	Comfortable making clinical decisions under pressure	3	3–4	4	4–4
Q4	Can communicate decisions with team under stress	4	3–4	4	4–5
Q5	Understands the role of triage in MCIs	4	3.5–4.5	5	4–5
Q6	Feels prepared to participate in disaster response	3	3–4	4	4–4.5
Q7	Can work efficiently in an interprofessional team	4	4–4	5	4–5
Q8	Understands ethical considerations of triage in disasters	4	3–4	5	4–5
Q9	Confident performing rapid assessment under pressure	3	3–4	4	4–5
Q10	Able to quickly identify life-threatening conditions	3	3–4	5	4–5
Q11	Recognizes importance of speed/accuracy in assessment	4	3–4	5	4–5

Open-ended survey responses (n = 23) were analyzed to explore students’ perspectives on the mass casualty incident simulation within the nursing curriculum. This analysis revealed four main themes and eight subthemes describing students’ experiences of the simulation (Fig 1).

**Fig 1:**
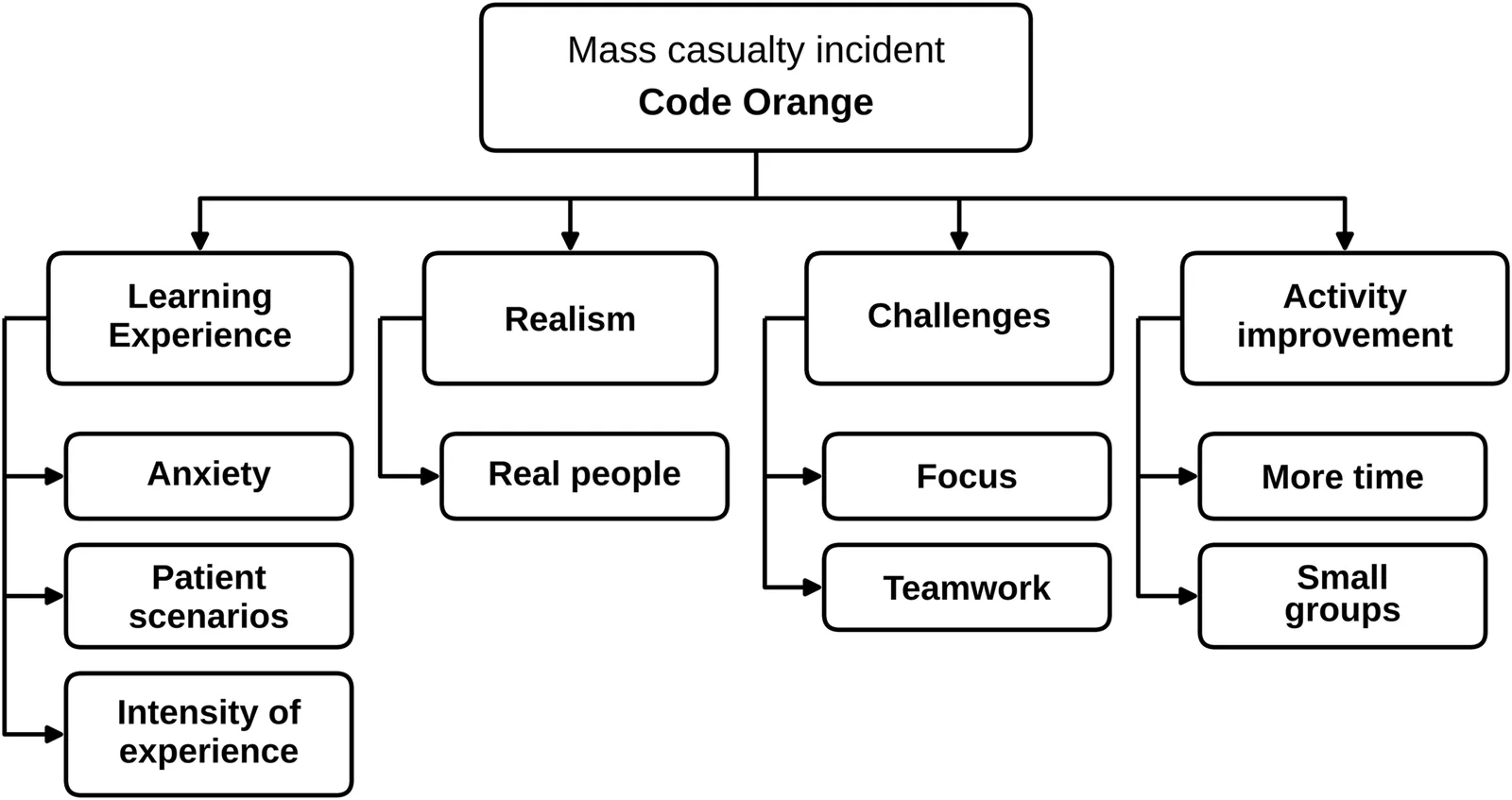
Coding tree of Themes and Subthemes Identified in the Code Orange Simulation.

### Learning experience

Students reported highly positive perceptions of the Code Orange mass-casualty simulation, describing it as a *“great learning opportunity”*, *“extremely beneficial,”* and *“highly realistic.”* Many emphasized that the immersive, high-fidelity environment contributed meaningfully to their learning, particularly through exposure to diverse patient presentations and dynamic scenario progression, which they felt enhanced their overall experience.

A prominent subtheme was the intensity of the experience, with several students noting the emotional and cognitive pressure inherent to the scenarios. This included feelings of initial anxiety, as expressed by P21: *“I felt a bit anxious at first,”* and difficulty managing competing stimuli, highlighted by P20: *“I felt overwhelmed because of the loud noises and acting. This impacted my decision-making because it was more pressure.”* Despite these stressors, students perceived the emotional intensity as a valuable component of the simulation, contributing to a more authentic understanding of triage demands during mass-casualty events.

### Realism

Most participants (n = 19) highlighted the strong sense of realism created by the simulation. Several students reported initially expecting to practice with mannequins; however, the incorporation of volunteer actors, environmental cues, and sensory effects exceeded their expectations and contributed to a more immersive learning environment. As P10 noted, *“I was expecting to only be interacting with simulation dolls; therefore, the use of real actors made it feel more real.”* Similarly, P11 emphasized the quality of the performance, stating, *“The volunteers did an incredible job acting their role and staying within the simulation, contributing to the learning environment being engaging and realistic”.* Participants also commented on the impact of auditory and atmospheric elements, with P13 sharing, *“I really liked how realistic it felt with the noises and smoke.”* These combined features enhanced the authenticity of the experience and supported students’ understanding of the sensory demands inherent in mass-casualty environments.

### Challenges

Students also identified several challenges experienced during the simulation, most of which were related to managing the high sensory load inherent in a mass-casualty environment. Participants described difficulty maintaining focus due to *“so much external stimuli”* and *“many things going on at once.”* The combination of noise, environmental cues, and physical conditions was frequently cited as distracting, with some students noting the environment felt *“very hectic and warm.”* Despite these challenges, several participants reported adapting over time. As P13 reflected, *“I was able to focus on the patient after the initial intake of the smoke.”* This suggests that while the sensory intensity was initially overwhelming, it ultimately encouraged learners to refine their ability to prioritize and concentrate under pressure.

Teamwork also emerged as a challenge. One student noted that *“working as a team can sometimes be just as confusing if organization was not prioritized,”* highlighting the importance of structured coordination in emergency response. However, participants also acknowledged positive aspects of team functioning, describing moments where team members supported one another and worked collaboratively to manage the demands of the scenario.

### Activity improvement

Students identified some opportunities to enhance the simulation. A key concern was the limited number of roles, which restricted active participation. To address this, they recommended reducing group sizes from six to approximately four students, allowing “each student to equally participate,” as one participant noted. Students also suggested extending the simulation time, noting that additional time would not only support deeper engagement and reflection but also enhance the intended stress and urgency characteristic of a mass-casualty incident, thereby reinforcing the realism and learning objectives of the activity.

## Discussion

The present study investigated the impact of a high-fidelity Code Orange simulation on nursing students’ knowledge, confidence, and preparedness for mass casualty incidents (MCI). The findings provide compelling evidence for the value of immersive simulation in disaster preparedness education and offer important implications for nursing curricula in Canada.

The primary finding of this study is the significant improvement observed in students’ CTAS/GCS knowledge, confidence, and overall preparedness following participation in the Code Orange simulation. This outcome aligns with established educational theories that emphasize the effectiveness of experiential learning in developing complex clinical skills and non-technical competencies [18]. The realistic scenario served as a powerful platform for students to actively practice decision-making, teamwork, and communication, skills that are critical yet difficult to teach in traditional classroom settings.

The high degree of realism and engagement achieved through environmental cues, such as noise, the use of actors, and realistic injury moulage, was a key factor in the simulation’s success. This high-fidelity design increased student immersion, which is known to support deeper, more active learning, and increase student’s confidence by bridging the gap between theoretical knowledge and practical application [19]. In addition, simulations that incorporate stressful scenarios in high-stakes environments may optimize clinician’s stress responses in real-world situations [20]. This was perceived by participants as they reported stress and emotional reactions during the simulation. Even though these emotional responses underscore the simulation’s effectiveness in replicating the psychological pressures inherent in MCI response, it is also discussed in the literature the negative influence of evaluation and observation during student’s performance on their stress levels [20]. Therefore, it is important that students are also prepared for the cognitive and emotional demands of a real-life Code Orange event during such simulations. The preparation should consider structured pre-briefing and debriefing processes that ensure psychological safety and sets clear expectations, while a comprehensive debrief allows students to process their emotional reactions, reflect on their performance, and consolidate learning, thereby mitigating potential negative psychological impacts. While those aspects have been incorporated in the simulated event presented in this study, Takhdat et al. [21] also suggests that incorporating interventions to support students coping strategies such as brief mindfulness should be considered to achieve robust learning outcomes in disaster education.

The findings from this study directly address ongoing gaps in Canadian nursing programs related to MCI and disaster training. While multiple Canadian hospitals and health-care institutions regularly conduct disaster and patient deterioration simulations, there is a distinct lack of evaluated simulated experiences embedded within post-secondary nursing curricula [22]. Given the demonstrable learning benefits, programs should consider integrating disaster simulations more regularly and progressively, moving from low-fidelity to high-fidelity scenarios. This progressive integration would ensure students are systematically exposed to the complexities of disaster response throughout their education, building competence incrementally. The successful implementation of this Code Orange simulation provides a blueprint for other Canadian nursing programs seeking to enhance their disaster preparedness education and details on its development will be shared in a separate publication.

### Study limitations and recommendations for future research

This study is subject to some limitations. The use of a single-site sample and voluntary participation may limit the generalizability of the results. In addition, the sample consisted largely of mature students with greater life and prior learning experiences, which may further limit the generalizability of the findings to other undergraduate nursing student populations. Furthermore, the reliance on short-term data means the long-term retention of knowledge and skills remains unknown. To build upon these initial findings, several recommendations for future research are proposed. Larger, multi-site studies are needed to validate the findings across different institutional and regional contexts. Long-term follow-up studies are essential to assess the durability of the acquired knowledge and confidence. The development and evaluation of interprofessional disaster simulations involving nursing, medicine, and other allied health professions would better reflect the collaborative nature of real-world MCI response and should be a priority for future educational research. No a priori sample size or statistical power calculation was performed, as this was a single-cohort pilot evaluation of a newly developed educational simulation rather than a hypothesis-driven efficacy trial. With only 23 matched participants, the study was likely underpowered to detect small-to-moderate effects, and several non-significant comparisons may reflect insufficient statistical power rather than a true absence of effect (Type II error risk). Effect sizes, where calculable, were modest to large only for the CTAS Level 2/3 vital-signs item; findings should therefore be interpreted as preliminary and hypothesis-generating pending replication in adequately powered samples. Additionally, the questionnaire used to assess knowledge, confidence, and preparedness was not previously validated, and formal psychometric properties (e.g., internal consistency, construct validity) were not established, which may affect the precision and interpretability of the self-report findings.

A related limitation is the likely presence of ceiling effects in the pre-test knowledge measures. Baseline CTAS and GCS knowledge scores were already very high before the simulation, with several items showing near-perfect pre-test performance. This ceiling constrained the range within which post-test improvement could be observed and may explain why most knowledge items showed no statistically significant pre-post change, independent of whether the simulation itself was effective. Future evaluations should consider measures with greater discriminative range at baseline (e.g., more complex case-based items) or recruit samples with more heterogeneous baseline knowledge to better capture the simulation’s educational impact.

## Conclusion

The Code Orange simulation effectively reinforced nursing students’ understanding of CTAS principles and disaster response processes, while also enhancing their self-confidence and perceived preparedness. Integrating disaster simulations into nursing education can significantly strengthen students’ competence in emergency and mass-casualty situations. The findings of this study highlight the value of such experiential learning in preparing nursing students for real-world clinical demands. Further exploration and refinement of the Code Orange simulation, specially through problem-based learning and structured skill development, may continue to support the growth of resilience, critical thinking, and confidence among future nursing professionals as they navigate high-stress environments.

## Appendix A: Debriefing Questions

- How do you feel about being involved in a disaster after going through this simulation?
- What went well?
- Was there any information missing that you would have liked to know, how would you obtain it?
- What would you do differently in the future if needed?
- Any areas that you feel you need more knowledge about?
- How did you feel after taking part in this simulation and having to assign CTAS/GCS scores to the patients? Is this your responsibility as a nursing student?
- Can you think of any ethical issues that could potentially arise?
- What are some take away points?

**Reference:** Bajaj K, Meguerdichian M, Thoma B, Huang S, Eppich W, Cheng A. The PEARLS healthcare debriefing tool. Academic Medicine. 2018;93(2):336. https://debrief2learn.org/pearls-debriefing-tool/

## Appendix B: Simulation Scenario Examples

- Patient 1: Debris in Eyes

20-year-old biological male/female. Victim walking/pacing and scared stating “I can’t see anything” often and loudly. Holding hands on and off face, complaining of pain in both eyes rates 6/10. The actor is to state they feel something in their eyes and occasionally rubbing soot off their face. Small amount of soot noted in and around Victims’ eyes. No difficulties breathing, breathing normally, awake, alert and orientated. Victims know their name, the current date, time and their current location (hospital parking lot) and obeys all commands from the nursing students. Vital signs normal or upper ends of normal due to pain. Eyes opening spontaneously with occasional squinting and rapid blinking even when not being spoken to, but the victim reports pain when trying to open and close eyes. Vital Signs: Temp 36.8 degrees Celsius, Pulse 98 bpm, RR 19 bpm, BP 140/80, 02 sats 97% on RA. GCS = 15 – eye opening response (Spontaneously = 4), Best verbal response (Oriented to time place and person = 5), best motor response (obeys commands = 6), CTAS 3 = urgent Could potentially lead to a serious problem requiring intervention, associated with significant discomfort, affecting the ability to work and activities of daily living. VS normal or upper ends of normal.

- Patient 2: Semiconscious Burn Victim

18-year-old biological male/female victim who is semiconscious located on a stretcher with a c-spine collar. Burns on 18% of their body (left and right arms). Unstable vital signs, breathing is spontaneous has shallow irregular breaths. O2 sats are abnormal and the patient has a weak thready pulse. The patient is moaning and groaning opening eyes to pain, localizes to pain and bystanders reported that the patient was thrown by the blast. Vital Signs: Temp 35.7 degrees Celsius, Pulse 121bpm. Respiratory Rate 30bpm, BP 71/50 mmHg, O2 sats 79% on RA, breathing spontaneously, shallow irregular breaths noted. GCS = 9 semi-conscious= (Eye opening response - opens eyes to pain =2, Best verbal response – moans and groans =2, Best motor response – moves to localized pain = 5) CTAS = 1 obvious signs of distress, unstable VS, immediate risk of deterioration, requiring immediate aggressive treatments (2nd to be seen, resp distress, low GCS).

- Patient 3: Hearing Loss and Bloody Nose

30-year-old biological male/female. Victim walking and scared stating “I can’t hear anything” often and loudly. Bloody nose noted when nurses assess. The victim rubs nose to wipe off blood and eyes to be able to see. A small amount of soot was noted in and around eyes. No difficulties breathing, awake, alert and orientated. Eyes opening spontaneously only when Victim visualizes, they are being spoken to. Victim reports no pain (mention this only if students find alternate means to communicate to Victim since the Victim can’t hear well). If trying to speak without alternate means of communication can state “I can’t hear what you are saying and appear frustrated”. The Victim knows their name, date, time and their location (hospital parking lot) and obeys all commands from the nursing students. This comprehensive portrayal will help the actor authentically represent a frightened, disoriented NOT confused Victim with hearing and mild vision issues following a traumatic event. Vitals Signs: Temp 36.8 degrees Celsius, Pulse 75 bpm, RR 18bpm, BP 137/89, O2sats 96% on RA. GCS = 15 (Eye opening response - spontaneously =4, Best verbal response – Oriented to person place and time = 5, Best motor response – Obeys commands= 6). CTAS = 4 less urgent. An acute issue with mechanism of injury (if chronic would be CTAS 5), would benefit from intervention or reassurance in 1-2 hour.

- Patient 4: Leg Impalement

60-year-old biological female/male. Victim sitting on ground against a building unable to move leg debris sticking out of mid femur of leg. Alert and oriented able to state date, time, name and location (hospital parking lot), eyes open spontaneously even if not being talked to. Able to control bleeding with tourniquet in place on leg. The victim is unable to walk. Minor scrapes and cuts noted on arms and face. Victim reports peripheral pain 10/10 – pain in leg and lower leg extremity. The victim is feeling very anxious. If students try to move or touch the victims wound they are to scream loudly in pain, to breathe a little faster than normal due to pain, but not to report any difficulties with breathing. This scenario provides a clear and realistic portrayal of a victim suffering from a blunt leg injury with debris sticking out of their leg. The victim is in severe pain and in shock. Vitals Signs: Temp 36.4 Degrees Celsius, Pulse 119bpm, Respiratory Rate 22bpm, BP 85/50 mmHg, O2 Sats 89% on RA. GCS = 15 (Eye opening response – spontaneously to speech =4, Best verbal response – Oriented to person place and time = 5, Best motor response – Obeys commands= 6). Alert oriented x 3. CTAS = 1 Emergent. Major trauma-blunt with obvious injury, conditions are threat to life or limb, don’t know if occluding artery. This patient requires rapid medical intervention.

- Patient 5: Small Abrasions to Hands

The victim is a 38-year-old biological male/female who is alert and oriented to person, place, and time (x3). They are able to recognize their name, the current date and time, and their location. The victim is speaking normally, although they are occasionally tearful due to the trauma of witnessing the explosion. At the time of the incident, the victim was exiting their car during the explosion and suffered minor abrasions to their left hand from glass debris. After exiting the car, the victim recalls running to assist others. The victim is a staff member who was preparing to start their shift at the time of the event. Although they are emotionally shaken from witnessing the accident, they have expressed that they do not wish to be triaged and prefer to go directly to the hospital so they can begin their shift. Vital Signs: Temp 36.6 degrees Celsius, Pulse 75 bpm, Respiratory Rate 14, BP 120/58 mmHg, O2 sats 98% on RA. GCS = 15. GCS 15 – Eye opening response – spontaneously to speech = 4, oriented x 3 person place and time = 5, obeys commands 6 Pain 1/10 - small abrasions from glass debris on hands and face – patient states they were getting out of the car when the blast occurred were located far away from the blast and then ran to help others on scene. CTAS 5 – non-urgent. Without evidence of deterioration. Investigations or interventions could be delayed or even referred to other areas of the hospital or health care system.

- Patient 6: Deceased Patient

A 35-year-old male thrown onto the sidewalk from the blast, was closest to the explosion. Vital signs absent. Pupils unresponsive. Burns noted on right side of the patient’s body. GCS = 3 currently unconscious (Eye opening 1 – No eye-opening to any stimulus. Verbal response 1 – no verbal response. Motor response – 1 no movement even to painful stimulus). CTAS 1 – requiring resuscitation (CPR to begin on scene). If the resources are available, will start CPR and bring into trauma. If no resources available, the person would be left (e.g. War zone), so that the resources could be allocated to support those who have an increased chance of survival but initial attempts to secure airway and breathing would be provided.

## Supporting information

S1 Checklist STROBE statement: checklist of items that should be included in reports of observational studies. (DOCX)

S2 Dataset Aggregate response data underlying the quantitative findings. (XLSX)
